# Reduction of the Dimensionality of the EEG Channels during Scoliosis Correction Surgeries Using a Wavelet Decomposition Technique

**DOI:** 10.3390/s140713046

**Published:** 2014-07-21

**Authors:** Mahmoud I. Al-Kadi, Mamun Bin Ibne Reaz, Mohd Alauddin Mohd Ali, Chian Yong Liu

**Affiliations:** 1 Department of Electrical, Electronic & Systems Engineering, Faculty of Engineering and Built Environment, Universiti Kebangsaan Malaysia (UKM), Bangi, Selangor 43600, Malaysia; E-Mails: mamun.reaz@gmail.com (M.B.I.R.); mama@eng.ukm.my (M.A.M.A.); 2 Department of Biomedical Engineering, Al-Khwarizmi College of Engineering, Baghdad University, Baghdad 47146, Iraq; 3 Department of Anaesthesiology & Intensive Care, UKM Medical Centre, Jalan Yaacob Latif, Bandar Tun Razak, Cheras, Kuala Lumpur 56000, Malaysia; E-Mail: chianyong@yahoo.com

**Keywords:** electroencephalogram (EEG) signal, anesthesia, surgeries, channels, signal processing, features, decomposition, criteria

## Abstract

This paper presents a comparison between the electroencephalogram (EEG) channels during scoliosis correction surgeries. Surgeons use many hand tools and electronic devices that directly affect the EEG channels. These noises do not affect the EEG channels uniformly. This research provides a complete system to find the least affected channel by the noise. The presented system consists of five stages: filtering, wavelet decomposing (Level 4), processing the signal bands using four different criteria (mean, energy, entropy and standard deviation), finding the useful channel according to the criteria's value and, finally, generating a combinational signal from Channels 1 and 2. Experimentally, two channels of EEG data were recorded from six patients who underwent scoliosis correction surgeries in the Pusat Perubatan Universiti Kebangsaan Malaysia (PPUKM) (the Medical center of National University of Malaysia). The combinational signal was tested by power spectral density, cross-correlation function and wavelet coherence. The experimental results show that the system-outputted EEG signals are neatly switched without any substantial changes in the consistency of EEG components. This paper provides an efficient procedure for analyzing EEG signals in order to avoid averaging the channels that lead to redistribution of the noise on both channels, reducing the dimensionality of the EEG features and preparing the best EEG stream for the classification and monitoring stage.

## 1. Introduction

The electroencephalogram (EEG) is a device used to determine the brain activity by electrodes placed in different places on the scalp. An EEG signal is a function of time and reported in terms of its frequency, amplitude and phase. Anesthesia is an irreplaceable portion of surgeries, where the anesthesiologists observe the depth of anesthesia (DOA) of patients based on monitoring the essential variations in physiologic indicators, such as eye movement, heartbeat, breathing rates and blood pressure, and their physical responses to excitation from the surgical procedure. The variation of the brain signal features are strongly related to the level of anesthesia; these variations are exploited to observe the DOA [1]. Unconsciousness is defined as lack of movement and unresponsiveness to painful stimuli, whereas the strength of operative stimulation depends on the duration and type of surgery [2]. Inappropriate general anesthesia, such as underdosage or overdosage, leads to intraoperative awareness or prolonged anesthesia. Under no circumstances, the common element that participates in appropriate general anesthesia is the ability to analyze the EEG signals to discern the stage of awareness, which leads to avoiding the risk of postoperative complications [3–5]. Scoliosis correction surgery is one of the most complicated operations that requires a long time under the influence of the anesthetic drugs (more than three hours). During these types of surgeries, surgeons use a lot of electronic devices and hand tools that increase the errors and noise level for the events of the EEG signal, such as neuromuscular monitoring, suction, cautery and X-ray. Most of these devices affects the channels that record EEG signals asymmetrically; they add DC voltage and noise to the recorded signal; thereby, we get a distorted wave and may not be able to it again [6,7]. In the same context, artifacts emerge from the patient, due to respiration, sweating, muscle activity, heartbeat and eye blinks [8–10]. The effect of these errors and noise on the EEG channels is discussed in detail in Section 3.3.

There are many methods used to reduce the dimensionality of the EEG signal, such as Principle Component Analysis (PCA), Independent Component Analysis (ICA) and Linear Discriminant Analysis (LDA). All of these methods are not used to reduce the dimensionality of the recorded EEG data; they are used to reduce the dimensionality of the features that are extracted from the whole recorded data. These methods transform the features to coefficients to another dimension according to the eigenvalues and eigenvector. For example, if the feature dimension is (*k* × *d*); the new dimension will be (*d* × *d*) [11]. These methods are different from each other in the way of finding the coefficients for the features. One of the main disadvantages of these methods is the possibility of losing important features that describe the variation of EEG signals, especially if the signal changes its component rapidly. In our proposed system, the features are used only to assess the components of the EEG signal without any reduction. This system reduces the number of channels according to the features without any transformation. The main advantage of the proposed system is to keep the important data, which enables us to predict the DOA, where we will not lose any of the components that make up the data. Certainly, this will be reflected in the features of the data and the accuracy of the classifier in the final stage.

After World War II, researchers looked after improving the classification of brain signals, which allowed them to diagnose abnormal waves. In the 1950s, William Grey developed the topography of the EEG waves, where he covered the mapping of the electrical activity of the human brain. From the 1980s, the initial stage of EEG data has been described by digital filters, which are used to remove the unwanted frequencies of the recorded signal. The chain of low–high-pass filters are used to remove the electrical line noise from the recorded EEG signal [12]. Nitschke *et al.* [13] reported that diagnosing the EEG signal in the time domain of the digital filter typically comprises cross-multiplication of each noisy datum and its neighbors with a set of weights. The discrete wavelet transform (DWT) is an efficient method used to denoise the acquired signals from various artifact waves that overlap with EEG signals, such as inherent noise, ocular artifacts and motion artifacts [14]. Other researchers combined the Wavelet (WT) and (ICA) to generate a new formula, Wavelet-Independent Component Analysis (WICA) technique. This technique exhibited the best performance to remove the artifacts from EEG signals with minimal information loss [15].

The aim of this paper is to analyze and test the recorded EEG signal during this type of surgery for choosing the channel that contains the minimum noise component. Averaging the EEG signal that comes from different channels leads to distributing of the noise that is carried by one channel to the other channel, which may worsen the errors in the EEG signal. This study adopted five stages to check the EEG channels. At the first stage, the recorded signals are filtered from the undesired noise and artifacts using four different techniques. The second stage is a Level 4 wavelet decomposition technique to decompose the signal of each channel to the details and approximation coefficients. The third stage tests these components by four different criteria (mean, energy, entropy and standard deviation). The fourth stage compares Channel 1 and Channel 2 to find the clean channel according to the criteria's value in stage three. The fifth stage acts as switching mode between Channel 1 and Channel 2 to generate a new combinational signal according to the results in stage four. To assess the results, the power spectral density, cross-correlation function and cross-coherence are used as the evaluation criteria. The results showed that the recorded signals corrected significantly without effectively changing the shape of the EEG signal, making it ready for extracting accurate features. This paper is arranged as follows: Section 2 describes the anesthetic agents and the recording succession. Section 3 describes the characteristics of the EEG wave bands and the effects of errors and noise to the channels that are used to record the EEG signals during surgery. Section 4 illustrates the stages of the proposed system, describing the evaluation criteria. Section 5 discusses in detail the experimental results. Finally, Section 6 presents the conclusions. This paper provides an efficient procedure to solve the problem of the EEG signal during a noisy surgery with long-term anesthesia.

## 2. Acquiring the EEG Data during Scoliosis Correction Surgeries

**Figure 1.**
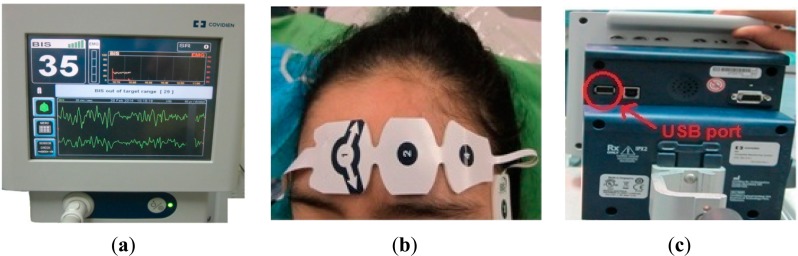
Depth of anesthesia (DOA) monitoring system by Covidien: (**a**) the screen of the monitoring system; (**b**) the distribution of the electrodes; and (**c**) the recording port.

Recording the EEG data during these types of surgery is somewhat difficult, because these surgeries are complex and take a long time (more than three hours), with a large number of staff in the operation room (four surgeons, three anesthetist and six nurses). Therefore, the data were acquired from six patients, which were sufficient to obtain good results in this research. Two channels of EEG data recorded using the DOA monitoring system by Covidien are shown in Figure 1a.

The four Zipprep electrodes, usually arranged diagonally on the patient's forehead to produce two EEG channels, are shown in Figure 1b. The electrode positions are arranged as follows: the common reference electrode at the center forehead (FPz) approximately five centimeters above the bridge of noise; the two channels directly above the eyebrow (FP2-AP8); and finally, the ground electrode on the temple between the corner of the eye and the hairline (FT10). The impedances of the electrodes should be below 5 Ω [16]. The USB port is used to export the data to a removable drive, as shown in Figure 1c.

### 2.1. The Characteristics of the Acquired Data

The EEG data were recorded in the PPUKM-Universiti Kebangsaan Malaysia Medical center. The raw EEG data file is a binary file containing unfiltered EEG data. The data are saved as received from the monitoring device; starting with the value of Channel 1, followed by the value of Channel 2, then a Channel 1 value, and so on. The recorded data were sampled at 128-times per second per channel; each EEG sample is represented by a 16-bit signed integer with units of 0.05 μV. The recording sequence of EEG signals is as follows: recording five minutes before giving the anesthetic agents; five minutes starting from the first moment of injecting of the drugs; 90 min during the surgery; and finally, ten minutes starting from the moment of stopping the anesthetic agents. The recorded data were presented in an ASCII code format; initially, these data were turned to hexadecimal form, then were converted to signed numerical formula. These data are processed using HxD-Hexeditor 1.7.7.0 to change the data from ASCII code format to the signed numerical formula, EXCEL 7 for statistical analysis and MATLAB 2012 to processes the EEG data.

### 2.2. The Anesthetic Agents

**Figure 2.**
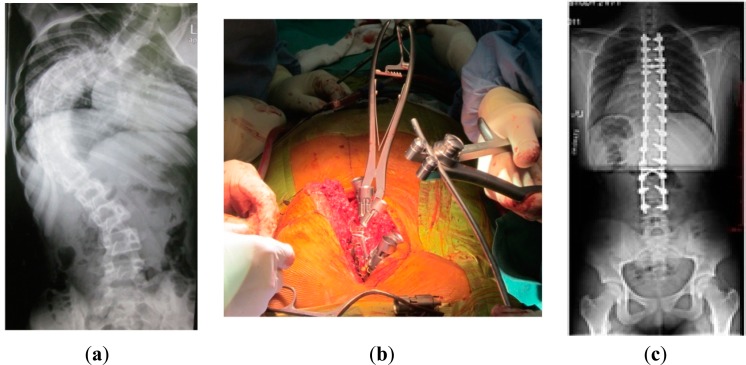
The spine: (**a**) before surgery; (**b**) during scoliosis correction surgeries; and (**c**) after surgery.

After obtaining the ethical approval, six patients who underwent scoliosis correction surgeries were recruited. Figure 2a proclaims the shape of the spine before surgery. Figure 2b shows the tools that were used during this type of surgeries that directly affected the EEG signals. Finally, Figure 2c illustrates the success of the scoliosis correction surgery.

When the patient takes the proper dose of intravenous anesthetic agents (changed to a deep unconscious phase), we stop recording and disconnect the socket of the electrodes. The patient is turned over, facing downwards, in order to be ready for surgery, then the socket of the electrodes is reconnected. The patients were classified as American Society of Anesthesiologist class I (ASA I), the age between “13–22” years with an average of 16.1 years, a weight between “31–42” kg with an average of 36.7 kg and a height between “127–153” cm with an average of 141 cm. All patients had general anesthesia with total intravenous anesthesia using target controlled infusion (TCI) of propofol and remifentanil, oral tracheal intubation and oxygen with air ventilation. TCI propofol using Schneider's protocol was set to 2–4 ng/mL and TCI remifentanil using Minto's protocol was set to 2–5 ng/mL. The Bispectrum Index (BIS) of the patients adjusted between “40–60”, as per normal anesthetic management.

## 3. The Properties of the EEG Signal

The shape and amplitude of the brain signal vary according to the level of anesthesia; also, the noise ratio varies from phase to phase during surgery. To analyze the EEG signals during the different stages of the surgery, we need to comprehend the characteristics of these signals, such as amplitudes, frequencies and internal and external effects that lead to a significant change in the form of the recorded signal.

### 3.1. Characteristics of EEG Wave Bands

As usual, ±100 μV is the dynamic range of the recorded EEG signal before amplification. Habitually, the brain wave is divided into five frequency bands according to the generators and rhythms [17,18].

1. Delta band δ: Thalamus generates this signal with a frequency range of 0.1 to 4 Hz and amplitudes from 20 to 200 μV. Usually, this band is associated with young patients, underlying lesions, encephalopathies and deep sleep.
2. Theta band θ: Customarily, this band is generated by neocortex and hippocampus with a frequency range from 4 to 8 Hz and amplitudes from 20 to 100 μV. This band is associated with childhood, adolescence, young adulthood and drowsiness.
3. Alpha band α: Sometimes known as Berger's wave, this band is generated by thalamus over the occipital (visual) cortex with a frequency range of 8 to 16 Hz and an amplitude from 20 to 60 μV. This band is so important, because it is related to the alert state of consciousness.
4. Beta band β: The cortex generates this band with a frequency range of 16 to 32 Hz with a low amplitude (2 to 20 μV). Rhythmic beta waves are associated with the active concentration, thinking, drug effects and various pathologies.
5. Gamma band γ: Some researchers classify these waves as beta band, because they have similar properties, but the frequency ranges from 32 to 64 Hz with very low amplitudes (3 to 5 μV).

### 3.2. The Variation of the EEG Wave during Anesthesia

The amplitude and frequency of the EEG signal is directly affected by increasing the concentration of the anesthetic agents. Other parameters also affect the brain signals, such as the patient's age, the type of anesthetic agent and the type of surgery. Firstly, lower doses of agents will decrease the amplitude of the alpha band and increase the amplitude of the beta band in the frontal regions. The artifact appears clearly at this stage due to the eye movement. Increasing the concentration of anesthetic agents to the surgical level leads to a decrease in the frequency of delta and theta bands. Further increases in the concentration of the anesthetic agents will affect the EEG signal and generate a special pattern known as burst suppression (BS). Alternating periods of high amplitude and low voltage are the main feature of this pattern. Any further increases in the dose of the anesthetic agents will cause a suppression and electrical silence. Finally, the induction of anesthesia associated with the frontal portion of the brain with increased beta activity and delta activity appears in the posterior regions and migrates toward the frontal regions [19–21].

### 3.3. The Effect of Errors and Noise on the EEG Channels

**Figure 3.**
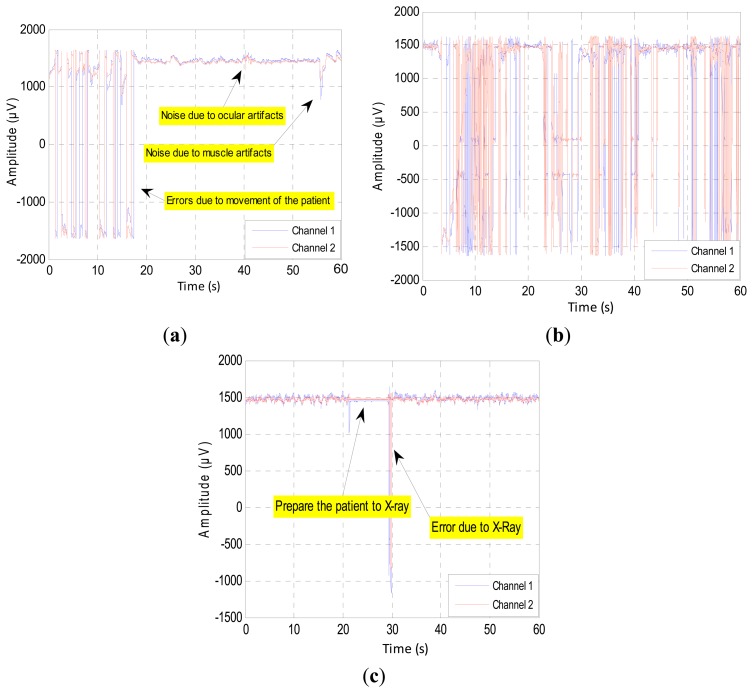
The occurrence of errors and noise due to: (**a**) patient movement; (**b**) hand tools and electric devices; and (**c**) high frequency electronic devices.

The severity of the errors varies from one channel to another according to the intensity of the intrinsic or extrinsic stimulation. Most of the bursts of errors occur during the surgery, due to extrinsic factors. During the operation, the patient is exposed to relatively violent movements, such as knocking, screw insertion and tightening of rods. These types of noise lead to change the value of some samples, because these samples overlapped with high-energy pulses, as shown in Figure 3a. This figure illustrates the occurrence of errors within the first 18 s due to the patient's movement. The second source of errors is the electric devices, like cautery, suction and neuromuscular monitoring. In this case, the EEG signals will disappear, because they are embedded in a stream of high power pulses, as in Figure 3b. Finally, the third source of errors is the high frequency electronic devices, such as X-ray. The surgeons use this device from time to time during these surgeries to check the position of screws and rods [22]. Here, we can estimate the EEG signal if the error samples are not as shown in Figure 3c. In all of these cases, the filters cannot perform their work, and the EEG signal appears fully distorted.

**Figure 4.**
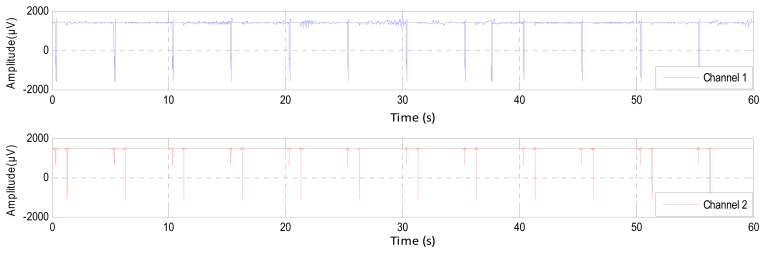
The synchronization of the noise and the strength of the artifacts in EEG channels during 60 s.

The inherent noise that emerges due to the electronic equipment can be eliminated by the high-quality electronic components of the EEG recorder. A shielded operating room can reduce the noise that comes from the high frequency electromagnetic devices [12,23]. In the same context, sometimes the EEG channels detect various kinds of artifacts that travel through different tissues. This contamination in the EEG signals appears as spikes and sharp waves overlapping recorded signals. The main reasons for artifact occurrence are involuntary actions, such as heartbeat, breathing, sweating, muscle activity and eye blinks. The sources that lead to the emergence of the noise are the same sources that led to the emergence of errors, but that are less in power, such as the movements of electrodes, head and eyes (EMG signal), as shown in Figure 3a. The electrodes are exposed to the same environmental conditions, but the noise and the artifacts will not affect the EEG channels homogeneously. The electrodes are affected according to the instantaneous impedance of the electrode or location and the intensity and type of the noise or artifacts. Figure 4 shows Channel 1 affected by the noise, while Channel 2 did not detect this noise in the same moment and *vice versa*. Furthermore, this figure shows that Channel 1 is affected by some artifacts, while Channel 2 detects these artifacts, but not in the same strength.

**Figure 5.**
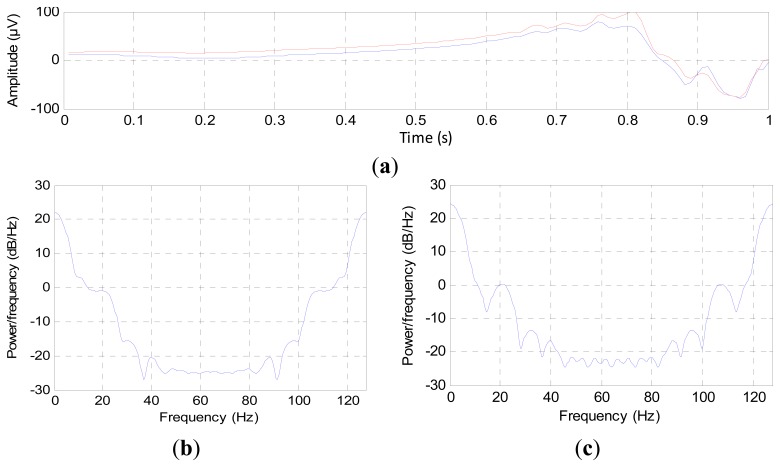
The power spectral of EEG channels: (**a**) EEG signals acquired by two channels; (**b**) power spectral density (PSD) for Channel 1; and (**c**) PSD for Channel 2.

The digital filters cannot remove the noise components that have the same frequency as the EEG signal, which is confined between 0.1 and 60 Hz; therefore, we must check the signal that is acquired from channels before using it to estimate the depth of anesthesia. To check the components of EEG channels, we used the power spectral density (PSD). The PSD values were calculated second by second for each channel, where this consists of two steps. The first step is normalizing the EEG signal by subtracting the mean of each second from the recorded EEG signal. The second step is finding the Fast Fourier Transform FFT to the normalized signal according to the sampling frequency. At first, we used only one second to demonstrate the difference between the channels, as shown in Figure 5.

Figure 5a represents one second of EEG signals for two channels; these signals are varying harmonically, but each channel carries different components. Figure 5b,c represents the PSD of Channels 1 and 2, respectively. From these figures, Channel 2 carries extra frequency components compared to Channel 1. This indicates that the channels have been affected by noise disproportionately [24].

A question that may be asked here is whether these additional components in Channel 2 represent EEG signals or noise? However, these components will never have a great effect on the estimation of the DOA, since we only need the general description of the EEG signal change in power and frequency, where we do not need the instantaneous changing of the EEG data. Therefore, we have to choose a channel of minimum components in order to decrease the processing time and to get accurate features that greatly reflect the DOA.

## 4. Methodology

**Figure 6.**
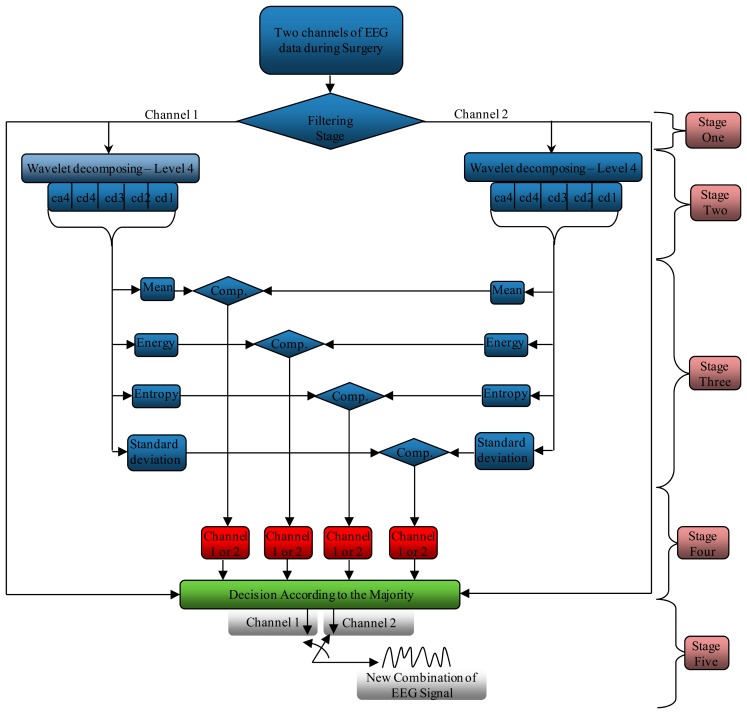
The stages of the proposed system for the comparison between the EEG channels to find the best channel representing the EEG signal.

As mentioned before, the noise does not affect the channels uniformly. The proposed system investigates the accuracy of the channels that were used to record the EEG signal, as well as choosing the channel that carries a lesser number of components. Any channel having fewer components indicates that the effect of the noise on this channel is less than the others. This system processes and tests the component of the signals for each channel separately, as shown in Figure 6. This system consists of five stages. The first one is a filtering stage, where it is undertaken to correct or delete the errors from the EEG signals and to denoise the acquired EEG signal. The second stage is dedicated to decomposing the signal using the wavelet decomposition technique to split the signal into several sections, according to the frequencies that contain it. In stage three, the system finds four different criteria (mean, energy, entropy and standard deviation) for each sub-band (ca4, cd4, cd3, cd2, cd1), where each channel is processed separately from another. The fourth stage is pinpointing the channel that carries minimum components according to the results that come from stage three. Finally, the fifth stage acts as a switching mode between Channels 1 and 2 to generate a new EEG signal from these channels according to the orders coming from stage four.

### 4.1. Filtering Stage

**Figure 7.**
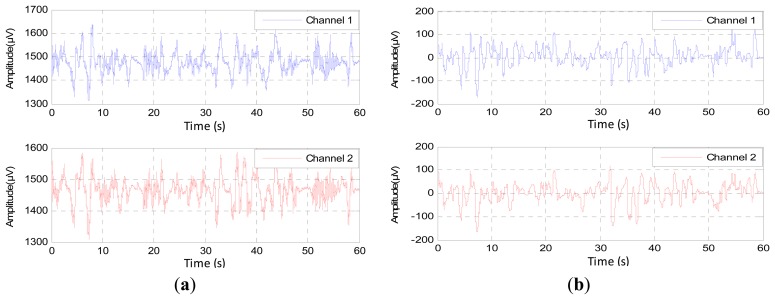
Sixty-second, two-channel of EEG signal: (**a**) unfiltered signal; and (**b**) filtered signal by the Band Stop Filter (BSF), Band Pass Filter (BPF), wavelet denoising technique (WT) and Savitzky–Golay filters (S-G Filter).

This stage is devoted to correcting and denoising the acquired EEG signals. This stage consists of four sub-stages: error correction, conventional digital filters, the wavelet denoising technique (WT) and Savitzky–Golay smoothing filtering (S–G Filter). The first sub-stage checks the error occurrence during the recording signal then corrects or removes the errors depending on the number of errors per second. If the errors are few and sporadic, the system can correct the error samples according to the previous samples. The system removes all 128 samples (one second) if a burst of errors occurs, because the EEG signals are embedded inside the error samples and the form of the original signal cannot be covered at all. The second sub-stage addresses removing the unwanted frequencies that appear in the EEG data using Band Stop Filter (BSF) or Notch Filter (NF) and Band Pass Filter (BPF). The notch filter removes the effects of AC line, while the band pass filters keep only the frequencies of the EEG signal that are confined between 0.1 and 64 Hz [25,26]. The third sub-stage is dedicated to removing the effect of the various kinds of artifacts, such as inherent noise, ocular artifacts and motion artifacts. The wavelet technique consists of three main steps. The first step is the decomposition the EEG signals to compute the wavelet coefficients (details and approximation). The second step is determining the threshold value from the coefficients that were founded in Step 1. To denoise the input signal, this technique resets the coefficients having an absolute value below the threshold level. Finally, the input signal is reconstructed by inverse WT according to the new coefficients. The mother wavelet “db4” function with Level 4 was used to remove the artifacts using wavelet denoising techniques (this will be explained in detail in the next section) [27,28]. The fourth sub-stage used the Savitzky–Golay smoothing filter to smooth out a noisy signal whose frequency span is large. To design this filter, we should define the order of the polynomial “*N*” and the frame size “*M*”, which represents the half width of the of the approximation interval [29]. Furthermore, the order of the polynomial must be strictly less than the frame size [30]. In this research, we used *N* = 17 and *M* = 33, because these values gave a good balance between the smoothing and denoising techniques. Figure 7a,b illustrates the original EEG signal and the filtered signal by these four methods, respectively. Apparently, the EEG signals are truncated from the noise, and ready to be sent to the next stage.

### 4.2. Wavelet Decomposition Technique Stage

This stage is dedicated to decomposing the filtered signal to provide sub-bands that represent the energy distribution of the EEG signal in time and frequency. This technique decomposes the EEG coefficients into the details and approximation coefficients. The approximation and detail frequency band of the DWT is directly related with the sampling rate and dominant frequency components of the original signals. The input EEG signal is decomposed into details and approximation coefficients into *N* + 1 sub-bands by an N-level WT. The sub-bands represent the approximation coefficient with the frequency band (0, *F*_S_/2*^N^*^+1^) and the sub-bands *d_j_* represent the detail coefficients with the frequency sub-band (*F*s/2*^j^*^+1^, *F*_S_/2*^j^*), where *j* = 1,…, *N*. The sub-bands corresponding to four decomposition levels for wavelet db4 with a sampling frequency of 128 Hz of the EEG signals are listed in Table 1. The signals were decomposed into details *d*_1_–*d*_4_ with one final approximation *a*_4_ [31,32].

**Figure 8.**
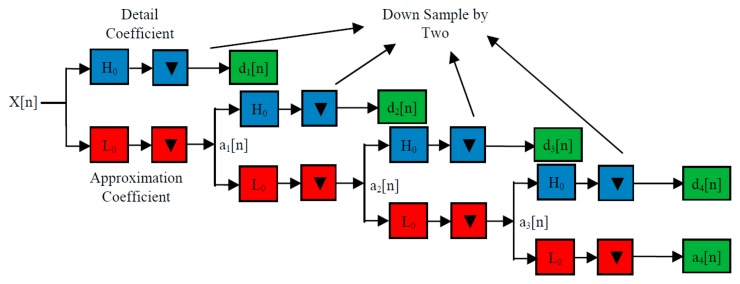
The four levels wavelet decomposition technique.

This decomposition was accomplished by a successive chain of low-pass and high-pass filters in a discrete time domain (adaptive filter). The input signal passes through a low-pass filter to get the approximation coefficients, and the same input is passed simultaneously through a high-pass filter to get the detail coefficients with a down sample of two at each stage, as shown in Figure 8 [27,33].

The samples of EEG signals are passed through a high-pass filter with impulse response *H*_0_; this convolution generates the detail coefficients *d*_1_ with a down sample of 2. The EEG signal also goes simultaneously through a low-pass filter with impulse response *L*_0_. The output of the convolution gives the approximation coefficients *a*_1_ with a down sample of 2. The approximation coefficients *a*_1_ also pass through a high-pass filter and a low-pass filter to generate the coefficients *d*_2_ and *a*_1_, respectively. This context will be continued, until we get the coefficients *d_N_* and *a_N_* [27].

This technique essentially depends on the suitable choice of a mother wavelet. There are a large number of mother wavelet functions; each one conforms to a specific application. If the form of mother wavelet function is chosen close to the form of the EEG signal, the WT process leads to the best possibility of energy localization in time domain. In fact, there is no well-defined rule for selecting a wavelet basis function in a particular application or analysis; some wavelet properties make a particular mother wavelet more acceptable for a given application and signal type [34,35].

**Figure 9.**
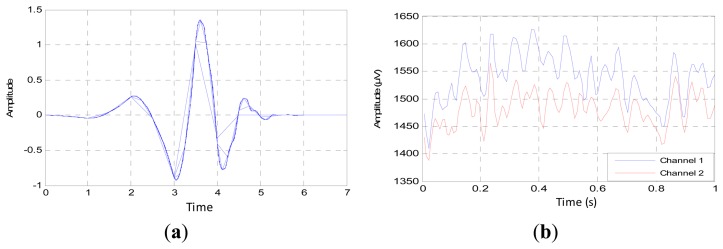
The compatibility between mother wavelet and the EEG signal: (**a**) mother wavelet “db4”; and (**b**) the variation of EEG signals.

Based on the previous general studies and our former study, the Daubechies function with the order of “db4” is a good mother wavelet to analyze the EEG signal, due to the optimality in the time-frequency localization properties, as shown in Figure 9a. Added to that, the waveform is so close to the recorded waveforms, as in Figure 9b [36–38]. From these figures, we can note clearly that the variance of the EEG signal has the same variation of the mother wavelet. This similarity will lead to correct decomposition and denoising of the EEG signal.

### 4.3. Processing Stage

**Figure 10.**
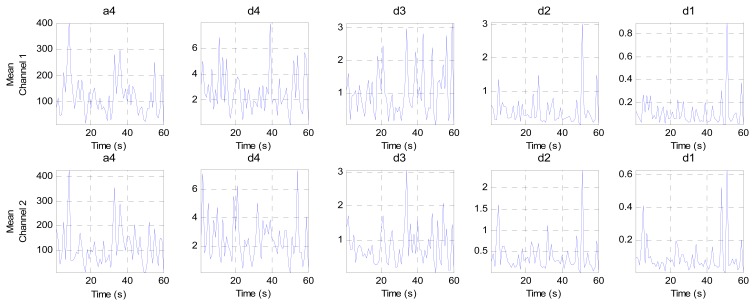
The mean values for two channels of the EEG signal sub-bands (a4, d4, d3, d2 and d1).

**Figure 11.**
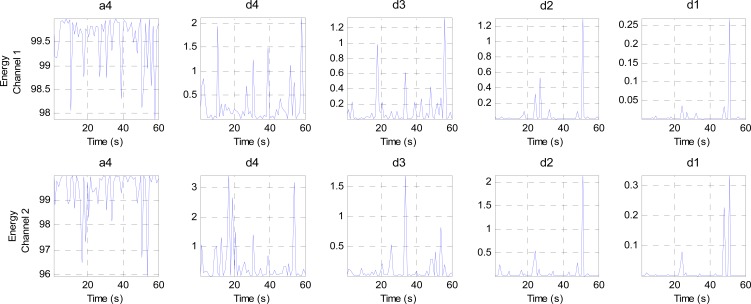
The energy values for two channels of the EEG signal sub-bands (a4, d4, d3, d2 and d1).

**Figure 12.**
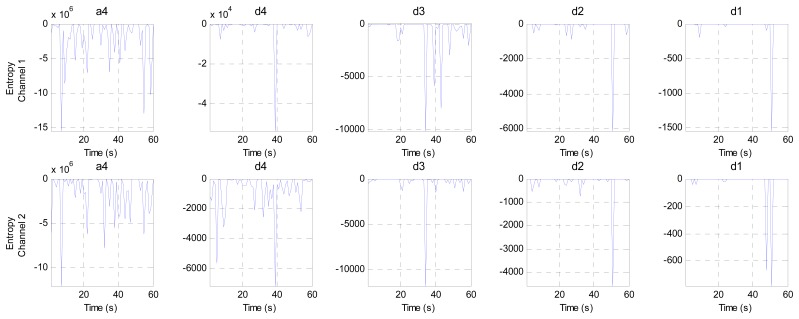
The Shannon entropy values for two channels of the EEG signal sub-bands (a4, d4, d3, d2 and d1).

**Figure 13.**
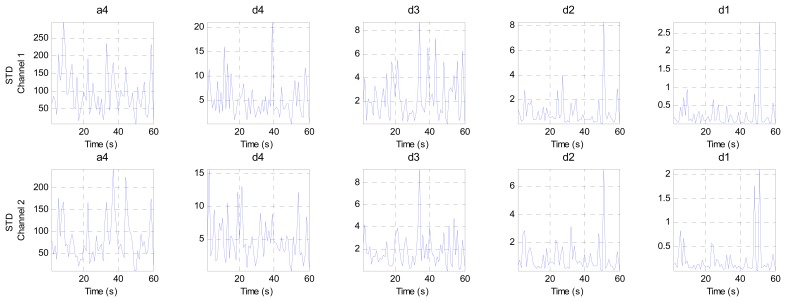
The standard deviation values for two channels of the EEG signal sub-bands (a4, d4, d3, d2 and d1).

This stage analyzes the contents of the sub-bands (ca4, cd4, cd3, cd2 and cd1) second by second to find the channel that has the minimum components (representing the best EEG channel). This stage consists of three steps. The first step calculates four criteria (mean, energy, Shannon entropy and standard deviation) for each band in both channels to extract the features from these bands, as shown in Figures 10, 11, 12 and 13, respectively. These figures represent 60 s of the EEG signal during the scoliosis correction surgery, which are decomposed by the wavelet technique and analyzed by the four criteria that were mentioned above. From this figure, we can note that the concentration of the frequencies varies from one band to another, where most of the power is concentrated within the frequencies, 0–4 Hz and 4–8 Hz. The concentration of power could change from one band to another based on two factors: the first depends on the level of anesthesia, while the second factor depends on the severity of the noise [39,40]. The second step is comparing the features of Channel 1 with the corresponding ones of Channel 2, where the upper band counts for the features that have the minimum absolute value. For example, the system compares (Mean-ca4-channel1) with (Mean-ca4-channel 2), (Mean-cd4-channel1) with (Mean-cd4-channel 2), and so on. Thus, we get five results for each criterion. The third step identifies the dominant channel within each criterion by comparing the results that got it in the previous step. The final results of the four criteria will be sent directly to the comparison stage.

### 4.4. Comparison Stage

**Figure14.**
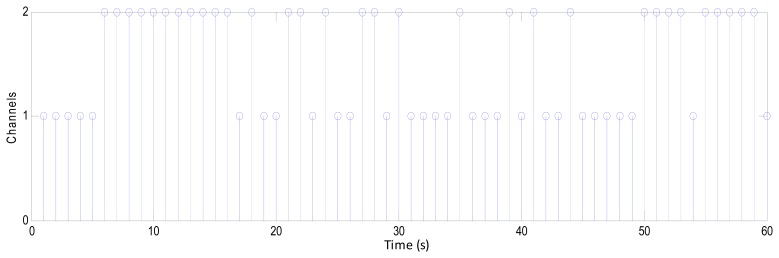
The last decision for 60 s of the EEG signal according to the status of the criteria.

This stage receives and compares the status of each criterion from the previous stage to identify the channel that carries the lowest compounds, giving the last decision. If the majority of the criteria tend towards Channel 1, the system sets this second as Channel 1, so if the criteria refer to the Channel 2, then the system sets this second as Channel 2, as shown in Figure 14. This figure represents the last decision for 60 s of the EEG signal, where the channels turn their positions according to the status of the criteria. This comparison is directly sent to the switching stage to generate a new stream of data.

### 4.5. Switching Stage

**Figure 15.**
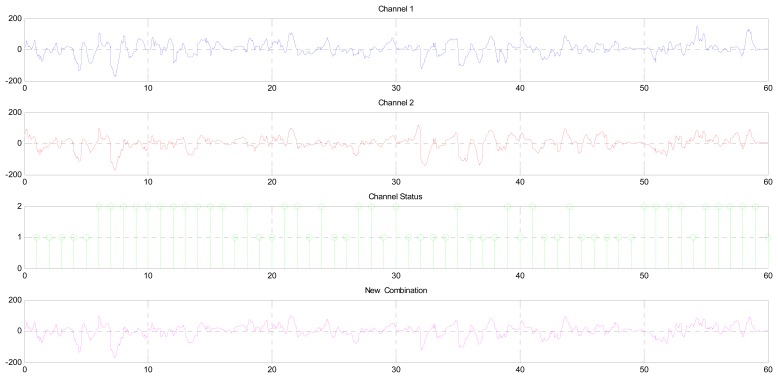
Combining the EEG channels: (**a**) 60 s of Channel 1; (**b**) 60 s of Channel 2; and (**c**) the new combination according to the status of the channel.

As we mentioned above, this stage will generate a new EEG signal from Channels 1 and 2 according to the status of these channels that has been received from the previous stages, as shown in Figure 15. This figure shows the EEG signal of Channels 1 and 2, the status of the channels (active or not) and the new combination.

This combination represents the final form of the EEG signal, and it is ready to be sent to the classification stage to monitor the depth of anesthesia. Finally, the system tests the feature of the channels; if both of the channels carry the same features, that means that there is no substantial noise or artifact overlapping with filtered signal and that the system can choose one of them. However, if the system detects an effective difference between the features of the channels, the system will choose the channel having the lowest components.

## 5. Experimental Results and Discussion

As we mentioned in the previous sections, our system consists of five stages for pinpointing the channel that carries the minimum components after filtering the EEG data during scoliosis correction surgeries. In this type of surgery, the surgeons use many types of electronic equipment and hand tools that increase the errors and noise, which directly affects the EEG signals. Moreover, some artifacts arise from the patient due to heartbeat, muscle activity, sweating, breathing and eye blinks. Practically, most of noise and artifacts are removed in the first stage, but some of the residual unwanted signals overlap with the original EEG signal, changing the amplitude of some samples or shifting the frequencies. It is impossible to identify the effects of such noise directly; only by the comparison between the components of EEG channels. If the channel component carries the same characteristics, this means that the noise did not affect the signal components drastically. However, if there is significant variation between the channel components, then we must compare between them and choose the lowest channel components (as shown in Figure 6 at the stages 2–5).

To assess the performance of the proposed system, we used 60 s of EEG signals for each patient at different stages of surgery. The experimental results were divided into three categories; power spectral density (PSD) [24,41], cross-correlation function (CCF) [42,43] and wavelet coherence (WC) [44,45]. To verify the proposed system in detail, these criteria were applied to the signals of Channel 1, Channel 2 and the combinational signal (the output of the system).

**Figure 16.**
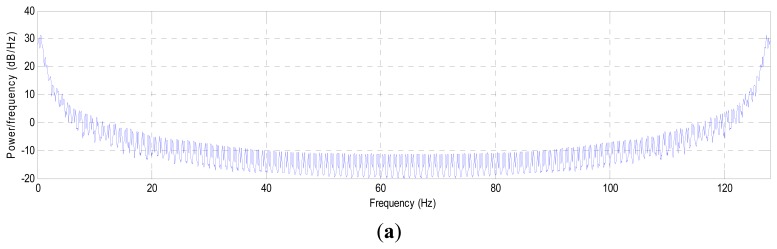

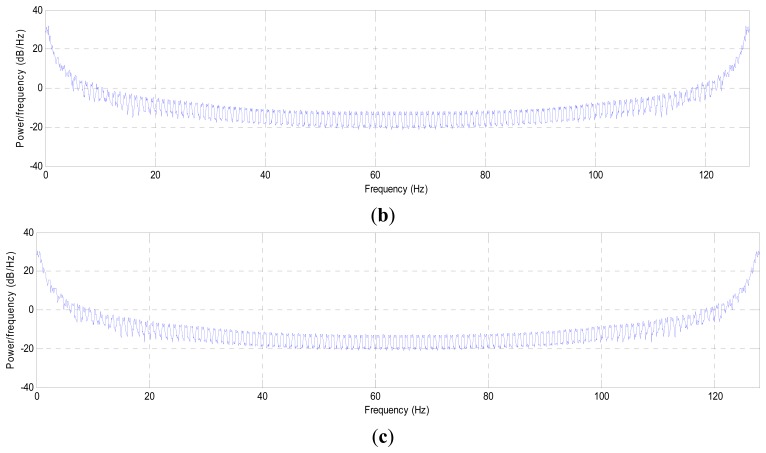
The power spectral density of: (**a**) Channel 1; (**b**) Channel 2; and (**c**) the combinational signal.

Returning to the first analysis of the signal, which is PSD, we notice that the combinational signal has the same power distribution of the original signal, Channel 1 and Channel 2, as shown in Figure 16a–c, respectively. These subfigures show that the emerging signal does not change drastically from the original signal. Added to this, this figure indicates that the combinational signal carries only the useful components of the EEG signals, which are disposed from the residual noise that overlapped with the original signal, which will lead to an increase in the calculation speed of the depth of anesthesia. In an example that is illustrated in Figure 16, most of the combinational signal is composed from the data that is coming from Channel 2, because they carry less components than Channel 1. We conclude from the above that the dimension of the data is reduced from two channels × 128 sample/s to one channel × 128 sample/s with pure EEG signals. This procedure reduced the processing time by half, with a signal carrying the most important components describing the variation of the EEG signal during anesthesia (without any residual noise).

Clinically, this process will reflect positively the idea of the DOA for many reasons; this process will increase the accuracy of the recorded EEG signal by selecting the most representative channel of the EEG signal changes during anesthesia, reducing the number of arithmetic operations that may contribute to cumulative errors and, finally, decreasing the processing time, where the DOA estimator will use only one set of unprocessed data (the combinational signal) to elicit the features.

**Figure 17.**
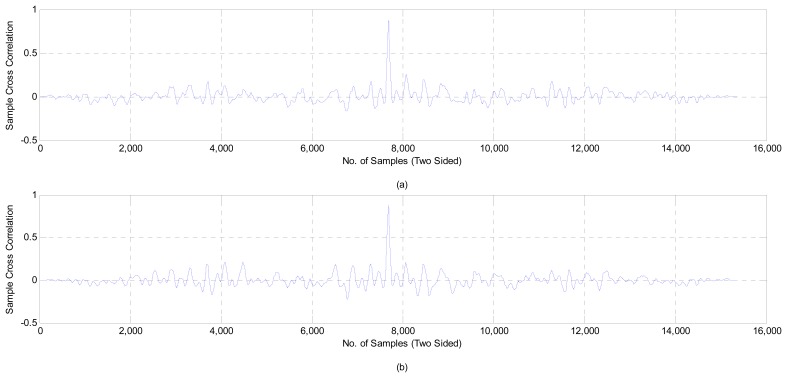
The cross-correlation function (CCF) between the signals: (**a**) Channel 1 and the combinational signal; and (**b**) Channel 2 and the combinational signal.

The CCF between the channel signal and the combinational signal is the second form that is used to verify the results, as shown in Figure 17. We all know that the double-sided CCF leads to the autocorrelation function (ACF) if the signal is tested by itself; that means that the average samples of the left side are equal to the average samples of the right side (the difference being equal to zero). Whenever the difference between the two sides is very little, that means that these signals are correlated with each other. Figure 17a shows the CCF between the signal of Channel 1 and the combinational signal that was found by the proposed system, while Figure 17b shows the CCF between Channel 2 and the combinational signal. These values give an indication that the output of the proposed system was correlated with Channel 2, which is consistent with the results that are shown in Figure 15.

To illustrate the result of CCF numerically, Table 2 shows the CCF for 60 s of the EEG signal during surgery; these signals were selected randomly from six patients. The second and third columns represent the number of times that each channel has been used in the resultant combinational signal, *i.e.*, these columns show the number of signals that are picked up from each channel to generate the combinational signal, because it carries the lowest component within 60 s. From these columns, Channel 2 is a prevalent channel for generating the combinational signal. The fourth and fifth columns show the difference between the average of the left and right side of the CCF of Channel 1, Channel 2 with the combinational signal, respectively, *i.e.*, finding the CCF of the channel (1 or 2)—combinational signal then we found (average (left side)—average (right side)). We notice that the values of the difference between the average of the left and right side of the CCF are different from patient to patient, according to the strength of the residual noise and artifacts that overlapped with the recorded signal. In all cases, this is chosen randomly; Channel 2 gave the lowest difference between the left and right side of CCF, which means that Channel 2 is the predominant channel for generating the combinational signal, because it contains less components than Channel 1. These results confirm the results of PDS and prove the accuracy of the proposed system.

**Figure 18.**
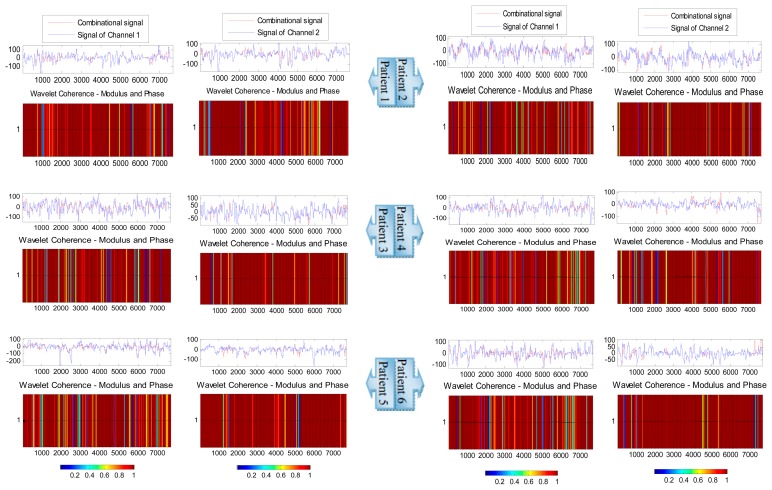
The analyzed signal–wavelet coherence modulus and phase between the combinational signal and Channels 1 and 2.

Finally, to assess the performance of the proposed system, sample by sample, the wavelet coherence (WC) is used to follow the variation of the combinational signal with respect to Channels 1 and 2 [46]. Figure 18 illustrates the coherence (analyzed signal–wavelet coherence–modulus and phase) of the combinational signal with Channels 1 and 2 by testing one second of EEG signal for six patients.

The arrows in the figures represent the relative phase between the two signals as a function of scale and position. The plot of the relative phases is superimposed on the wavelet coherence. From this figure, we notice that the combinational signal is coherent with both channels, because it is derived from those channels, but the combinational signal is more coherent with the channel that carries less components (Channel 2). For example, at the modulus and phase figure of Patient 1, the combinational signal is coherent with both channels, because the number of times that have been used for Channel 1 are almost equal to the number of times used for the second channel, as mentioned in Table 2. The combinational signal is coherent with Channel 2 in the modulus and phase figures of the patients (2–6), because the second channel has the majority to generate the resultant signal. Moreover, from the modulus and phase figure, we can notice clearly that the combinational signal is in phase with the signal in Channels 1 or 2. From all of the above, the resulting signal does not change the shape or the phase of the original signal, which means that it is compatible with the changing of the EEG signals during different stages of anesthesia.

If we compare among these three criteria (PSD, CCF and WC), we find that the combinational signal reduces the dimensionality of the recorded signal without changing the signal properties, because it is completely derived from the original signal. This system is reliable for different kinds of surgery, because this system has been tested under noisy signals. As we mentioned before, this proposed system is completely different from the methods that were used for feature reduction, such as PCA, ICA and LDA. These methods change the whole signal to the features, then reduce the dimension according to eigenvalues and the eigenvector. Changing the whole signal to features will take a lot of processing time, and reducing the dimension of these features may lead to the loss of useful information.

## 6. Conclusions

EEG signals carry valuable information for the brain system. These signals are often contaminated with artifacts and noise arising from many sources. During scoliosis correction surgeries, the surgeons use several types of electronic devices and hand tools, leading to the increase of the noise level for the events of EEG signals. The noise does not affect the channels uniformly. This research proposed an automatic system to reduce the dimensionality of the errors and noise from the recorded EEG signal by investigating the accuracy of the channels that were used to record the EEG signal, as well as choosing the channel that carries a smaller number of components. Any channel having fewer components indicates that the effect of the noise on this channel is less than the others. The suggested system consists of five stages; a filtering stage (error correction, conventional digital filters, wavelet denoising technique (WT) and the Savitzky–Golay smoothing filter), the wavelet decomposition stage (Level 4), the processing stage to process the contents of the sub-bands (ca4, cd4, cd3, cd2 and cd1) by four different criteria (mean, energy, entropy and standard deviation), the comparison stage to identify the channel that carries the lowest number if compounds and giving the last decision and, finally, the switching stage to generate a new EEG signal from Channels 1 and 2 according to the status of these channels. Power spectral density (PSD), the cross-correlation function (CCF) and wavelet coherence (WC) were used to assess the proposed system. The experimental results show that most of (not all) the signal in Channel 2 is the best, and there is no significant change when it is combined with the signal of Channel 1 (no gaps, no delay and no distortion). Intentionally, we used the EEG signal prevailing for one of the channels in order to prove that the final signal is not different from the original signal, even after merging parts of the two channels. Furthermore, the experimental results show that the proposed system does not change the property of the recorded signals, and the combinational signal is completely compatible with the changing of the EEG signals during different stages of anesthesia. The reduced number of channels that are used to analyze the brain signal will lead to an increase of the speed and accuracy of the DOA indicator. Practically, the proposed system will convert only the most representative channel (combinational channel) to features. This technique increases the processing speed by reducing the number of arithmetic operations, which will be reflected in reducing the cumulative errors. This system provides an efficient procedure to solve the problems with the EEG signal during a noisy surgery with long-term anesthesia. If monitoring DOA becomes economic, safe and simple, all anesthesia cases can be monitored easily.

## Figures and Tables

**Table 1.** The sub-band frequency corresponding with the Level 4 wavelet decomposition technique.

Decomposition Levels	Frequency Bands	Frequency Bandwidth (Hz)
ca4	Delta	0–4
cd4	Theta	4–8
cd3	Alpha	8–16
cd2	Beta	16–32
cd1	Gamma	32–64

**Table 2.** The numerical analysis of cross–correlation function (CCF) for 60 s for each patient.

**Patients**	**The Channels during 60 s**		**The Difference between the Average of the Left and Right Side of the CCF**	**The Difference between the Average of the Left and Right Side of the CCF**

	Ch1	Ch2	Channel 1: combinational signal	Channel 2: combinational signal
P1	29	31	0.0103037	0.0030128
P2	21	39	0.0034	0.00064823
P3	16	44	0.0030	0.0026
P4	21	39	0.0056	0.0026
P5	15	45	0.0131	0.000076276
P6	15	45	0.0035	0.000 90878
